# Pipeline Inspection Gauge’s Velocity Simulation Based on Pressure Differential Using Artificial Neural Networks

**DOI:** 10.3390/s18093072

**Published:** 2018-09-13

**Authors:** Renan Pires de Araújo, Victor Carvalho Galvão de Freitas, Gustavo Fernandes de Lima, Andrés Ortiz Salazar, Adrião Duarte Dória Neto, André Laurindo Maitelli

**Affiliations:** 1 Departamento de Engenharia de Computação e Automação, Universidade Federal do Rio Grande do Norte, Lagoa Nova, Natal, Caixa postal 1524 CEP 59078-970, RN, Brazil; andres@dca.ufrn.br (A.O.S.); adriao@dca.ufrn.br (A.D.D.N.); maitelli@dca.ufrn.br (A.L.M.); 2 Instituto Federal do Rio Grande do Norte, Rua Antônia de Lima Paiva, 155, Nova Esperança, Parnamirim CEP 59143-455, RN, Brazil; gustavo.lima@ifrn.edu.br

**Keywords:** PIG, neural network, velocity measurement, microcontroller, testing pipeline

## Abstract

Industrial pipelines must be inspected to detect typical failures, such as obstructions and deformations, during their lifetime. In the petroleum industry, the most used non-destructive technique to inspect buried pipelines is pigging. This technique consists of launching a Pipeline Inspection Gauge (PIG) inside the pipeline, which is driven by the pressure differential produced by fluid flow. The purpose of this work is to study the application of artificial neural networks to calculate the PIG’s velocity based on the pressure differential. We launch a prototype PIG inside a testing pipeline, where this PIG gathers velocity data from an odometer-based system, while a supervisory system gathers pressure data from the testing pipeline. Then we train a Multilayer Perceptron (MLP) and a Nonlinear Autoregressive Network with eXogenous Inputs (NARX) network with the gathered data to predict velocity. The results suggest it is possible to use a neural network to model the PIG’s velocity from pressure differential measurements. Our method is a new approach to the typical speed measurements based only on odometer, since the odometer is prone to fail and present poor results under some circumstances. Moreover, it can be used to provide redundancy, improving reliability of data obtained during the test.

## 1. Introduction

Along the oil and gas production chain, the outflow of production is done mainly through pipelines due to the good cost-benefit relation (uninterrupted transport of product for long distances and costs concerning the payment of fees and infrastructure maintenance [1]).

Over the years, pipelines need to be inspected to detect possible defects such as corrosion, incrustations, kneading or any other that could reduce product flow. Inspections can be made by non-destructive techniques as the ones based on ultra-sound, X-ray and magnetic principles. However, these techniques typically analyze small areas each turn and are used in non-buried pipelines [2]. The oil and gas industry usually employs a Pipeline Inspection Gauge (PIG), which is a device that can quickly analyze long distances of pipelines, buried or otherwise, by sensors present on it [3].

Several researchers have developed works related to PIGs. For example, Tiratsoo [4] shows project details and analysis, basic theory and recommendations regarding pigging operations. Regarding the use of neural networks applied to pipeline inspections, Suna and Berns [5] develop an application of neural networks in smart PIGs called NeuroPipe, based on a self-organizing map, to detect failures on oil and gas pipeline. Carvalho et al. [6] and Ma and Liu [7] use magnetic flux leakage (MFL) data obtained from sensors present in smart PIGs to train and validate neural networks, with good results. Senouci et al. [8] develop a model that predicts the causes of failure in oil pipelines based on factors other than corrosion. Both models were able to predict pipeline failures due to mechanical, operational, corrosion, and natural hazards with an average validity of 90% for the regression model and 92% for the artificial neural networks (ANN) model. Li et al. [9] present a review about the application of neural networks to improve robots’ movement control used in inspection pipelines.

During typical operation, PIGs can reach speed values above the recommended range of its inspection sensors, which decreases the inspection performance, since they should ideally move at constant speed [10]. Therefore, the development of efficient velocity controllers is necessary, as well as velocity measurement systems for PIGs.

Hannifa and Hashim [11] state that the velocity control is requested for various types of PIGs and describe several related control techniques. Yardi [10] presents an extensive bibliography review of velocity controllers for PIGs and describes a control system that uses a bypass valve as the control actuator. Nguyen et al. [12] present a nonlinear controller that may be used to activate orifices inside the PIG in order to allow the passage of gas flux and, consequently, reduce the PIG’s velocity.

To calculate speed, PIGs typically use an odometer, which is prone to error due to its constructive characteristics. When the odometer loses contact with the internal wall of the pipe, due to a bumping into a mechanical defect, for example, an error in the measurement occurs. The magnitude of this error depends on factors like speed, size of defect and characteristics of the transported fluid [13].

Therefore, the purpose of this paper is to present the training of two neural networks to predict PIG’s velocity from pressure data on the testing pipeline and compare predictions with real data. This technique aims to provide redundancy to the typical velocity measurement systems based only on an odometer, improving the reliability of velocity data.

This work follows previous researches developed at Universidade Federal do Rio Grande do Norte (UFRN). An innovative technology to attenuate of PIG’s velocity peaks by using Fuzzy logic is addressed by Lima [14]. A supervisory system that uses pressure transducers installed on a test pipeline to calculate the velocity of PIGs is presented by Freitas [15]. Lima et al. [16] describe the validation of this system, comparing the velocity calculated by the supervisory with data gathered from the odometer. Finally, the use of neural networks to calculate PIG’s velocity from pressure differential is addressed by Araújo [17].

The next sections of this article are organized as follows: in Section 2, we present materials and methods. In Section 3, we show the results from training and validating two neural networks. In Section 4, we discuss the results. Lastly, in Section 5, the conclusions of this work are discussed.

## 2. Materials and Methods

### 2.1. Pipeline Inspection Gauge (PIG)

The PIG is a tool applied for pipeline inspection. It is launched inside the pipeline and moved by the action of the fluid transported with the objective of cleaning and verifying the integrity of the pipe [3,17].

There are three main types of PIG: cleaning PIGs, smart PIGs and special PIGs. Cleaning PIGs clean the pipeline interior, removing obstructions such as hydrates. Smart ones are able to analyze pipeline conditions. Finally, special PIGs are used in specific conditions, like when the interruption of certain sections of the pipeline is required [18].

**Figure 1:**
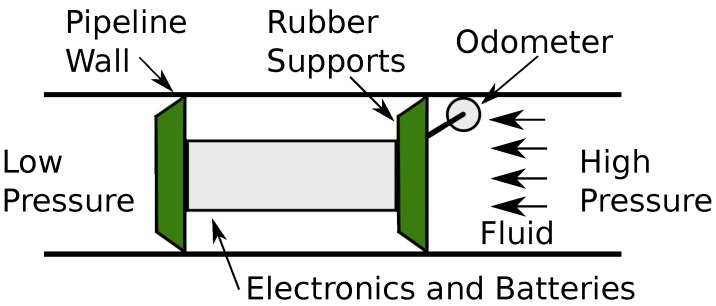
Example a PIG into the pipeline. Source: Lima et al. [16].

The structure of a typical PIG is illustrated in Figure 1. Isolated by rubber supports, the PIG’s body, generally made of rubber or metal, is where the electronics and batteries are arranged. The PIG moves through the pipeline due to pressure differential between the upstream (high pressure) and the downstream (low pressure) regions. The presence of the odometer is related to the measurement of some variables in smart PIGs (velocity, acceleration, travelled distance).

It is possible to model the movement of the PIG inside a horizontal pipeline applying the Second Law of Newton. Based on Nguyen [12,19] and Lima et al. [16], Equations (1) to (3) can be obtained:

(1) $$F_{p}-F_{a}=M\cdot a,$$

(2) $$F_{p}=\Delta P\cdot A,$$

(3) $$F_{a}=B\cdot v+F_{s},$$

where: $F_{p}$ is the force that causes the motion of the PIG due to the pressure differential ΔP applied on its backside area *A*; $F_{a}$ is the friction force between the rubber supports and pipeline wall, composed of viscous friction coefficient *B*, PIG’s velocity *v* and dry friction force $F_{s}$.

Substituting Equations (2) and (3) in Equation (1) and replacing $a=\dot{v}$, we obtain:

(4) $$\Delta P\cdot A-B\cdot v-F_{s}=M\cdot \dot{v}.$$

Isolating $\dot{v}$ and organizing the terms on the right side of equality, we obtain:

(5) $$\dot{v}\left(t\right)=-\frac{B}{M}v\left(t\right)+\frac{A}{M}\Delta P\left(t\right)-\frac{1}{M}F_{s}\left(t\right).$$

From Equation (5), it is verified that the PIG’s velocity depends mainly on the pressure differential.

### 2.2. Artificial Neural Networks (ANN)

Based on McCulloch and Pitts’ artificial neuron model detailed in *A logical calculus of the ideas immanent in nervous activity* article from 1943, the artificial neural networks (ANN) are mathematical models inspired on human brain and they present the capability of learning from examples [20].

**Figure 2:**
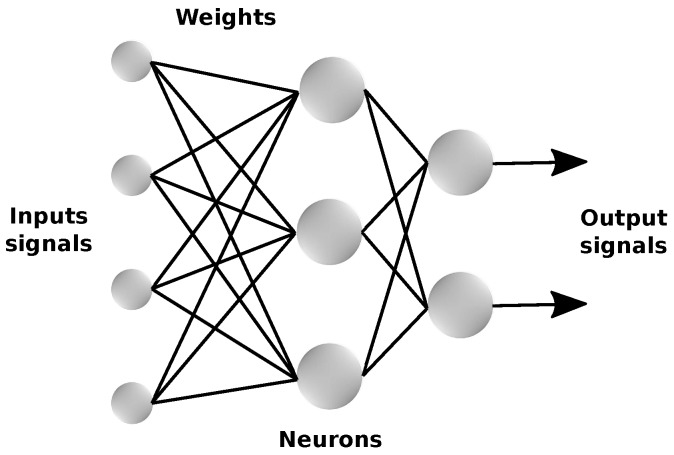
Basic structure of an artificial neural network. Source: Adapted from Oliveira [22].

As the biological nervous system, the ANNs consist of elements denominated neurons, which are interconnected and able to process information from external sources. In a neural network, illustrated in Figure 2, the input signals are multiplied by their respective weights and sent to every neuron of the following layer. The weighted signals are then added to a pre-determined value, called bias, which results in a value that will be used by the activation function to calculate the ANN’s output signal [21,22].

Mathematically, the representation of an ANN depends on the kind of model on which it is based. In general, the following equation may be used:

(6) $$y=\sum_{i=1}^{n}f\left(w_{i}\cdot x_{i}+b_{i}\right),$$

where: *y* is the ANN’s output signal; *n* is the number of inputs; *f* is the activation function; *w* is the ANN’s weight matrix; *x* is the ANN’s input signal; and *b* is the bias. The presence of the summation is explained by the fact that each neuron collaborates with the ANN’s output signal through its weight matrix.

Before an ANN can be used on any application, two steps must be taken: training and validation of the network. In the training step, both weight matrix and bias of each neuron are adjusted (from an initial value) until a minimum value of error (or other parameter) is reached. After training, it is important to validate the neural network by the application of input signals different of those used in the training step, to verify the generalization power of the trained network.

In this work, we have used two models of neural network: MultiLayer Perceptron (MLP) and Nonlinear Autoregressive with eXogenous input (NARX).

#### 2.2.1. Multilayer *Perceptron* (MLP)

**Figure 3:**
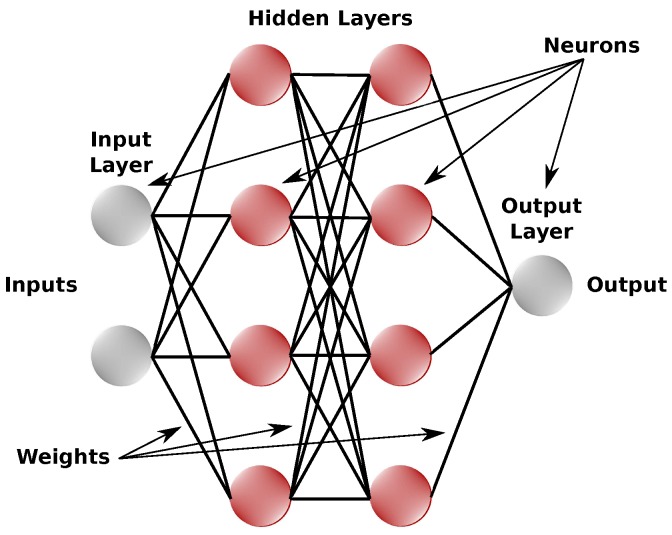
Example of MLP structure. Source: Adapted from Araujo [17].

In its more basic form, denominated *Perceptron*, an artificial neural network presents limitations when applied in real situations because of difficulty in minimizing the error function, mainly due to the existence of nonlinearities. One way to solve this problem is adding intermediary layers between the input and the output of the ANN. This architecture is called Multilayer *Perceptron* (MLP) [17], illustrated in Figure 3 below.

According to Haykin [21], the presence of intermediary layers, allied with the use of nonlinear functions in neurons, makes the network able to extract most significant characteristics from a data set, resulting on mathematic models more robust and more representative.

The MLP network has its training based on the minimizing of a cost function defined in terms of mean squared error. The basic algorithm is known as Backpropagation due to the way it acts: the input data (or signal) are applied in the entrance layer of the network and are propagated towards the output layer, after passing through each layer and interacting with each neuron along the way; at the output layer, the result signal is compared with the expected response, creating a value of error and, based on its value, the local gradient of the cost function; from this point, this error is propagated backwards from the output to the input layer, adjusting the neurons weights at each intermediary layer, along with the local gradient generated. The end of this process of adaptation of weights is reached when the cost function is reduced to desired levels.

#### 2.2.2. NARX Neural Networks

Although MLP networks can act in a great variety of real cases, there are situations in which the model obtained is not as good as required, despite its good response. Some of these cases are when temporal series are studied due to the fact that the current value depends on previous values, which impairs the learning of the correlation between input and output signals by ANN [17].

**Figure 4:**
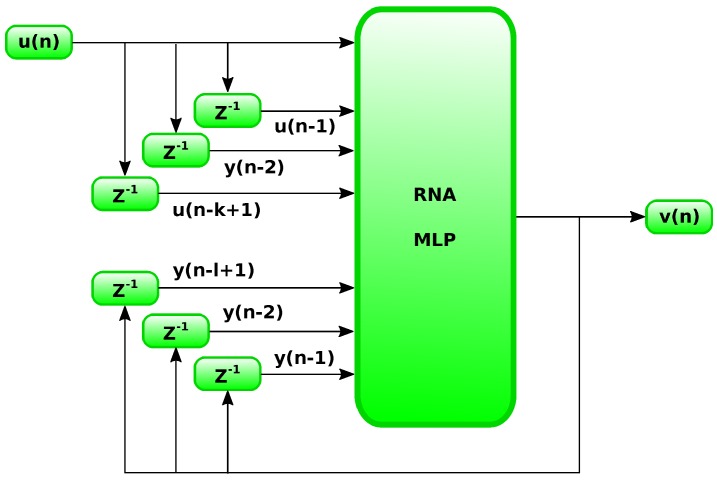
Example of NARX structure. Source: Adapted from Silva [23].

According to Silva [23], for treatment of temporal series, an ANN candidate is one based on NARX model, illustrated in Figure 4, which is a MLP whose network input is its own output y(n) feedbacked with temporal delay units $Z^{-1}$ and an input u(n) also delayed in time. Because the neural network input signal receives previous data, the behavior of data set is learned with more precision by the ANN, which leads to a more consistent model.

The training of a NARX neural network is performed the same way as a MLP network, using basically an algorithm to minimize the adopted cost function.

### 2.3. Methodology

**Figure 5:**
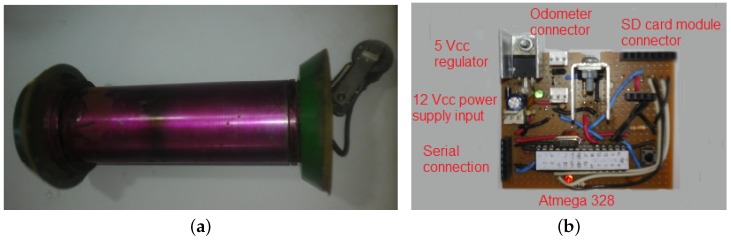
Devices used in this work: (**a**) Prototype PIG; (**b**) Electronic board. Source: By Authors.

The prototype PIG used in this work is presented in Figure 5a; its body is made with stainless steel and at the extremities, there are polyurethane-made rubbers. It includes an odometer at the backside and an empty space inside, where an electronic board and the battery are mounted. The total length of the prototype is about 50 cm and the diameter is 6 in.

The electronic board (Figure 5b) is composed of a microcontroller Atmega 328, an SD card module connector, a connector to the odometer, a 12 Vcc power supply input, a 5 Vcc regulator and a serial connection. The distance measurement is made by the odometer, which uses a Hall sensor inside the odometer’s wheel: for each complete turn made by the wheel, the Hall sensor sends a signal to the microcontroller; given the time between the consecutive signals and the length of the wheel, the PIG’s velocity is calculated.

**Figure 6:**
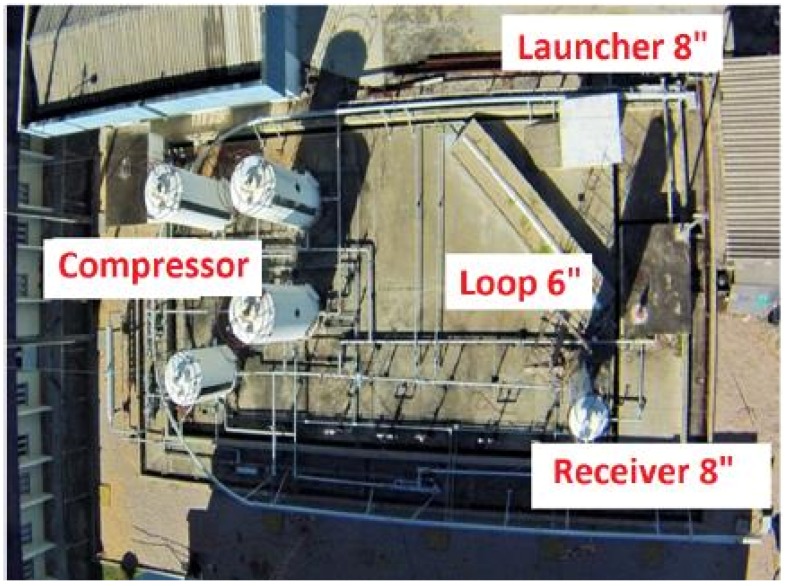
Testing Pipeline of 6” in Upper View. Source: Adapted from Lima et al. [16].

We have launched the prototype PIG in our test pipeline (Figure 6), which is located at UFRN. This pipeline is about 55 m long and thick of 5 mm, its structure features: an $8^{\prime \prime }$ diameter launcher, a straight section, a curved section, and then another straight section, all these sections with $6^{\prime \prime }$ diameter, and lastly an $8^{\prime \prime }$ diameter receiver. Besides that, a compressor pumps air to the interior of the test pipeline to propel the prototype PIG.

**Figure 7:**
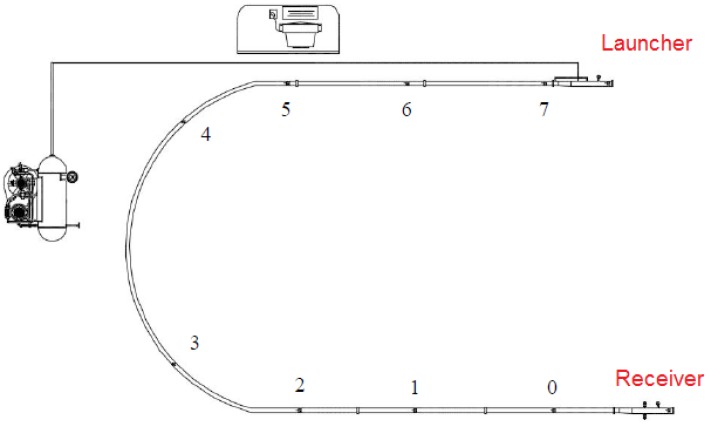
Positions of the pressure sensors along the pipeline. Source: By authors.

Along the test pipeline, there are 10 access points to take pressure readings. At the launcher and at the receiver, the pressure readings are taken with manometers. At the remaining eight points (Figure 7), the measurements are taken by NOVUS™ pressure transducers, model NP300, and then are sent to a Programmable Logic Computer (PLC), model WEG TPW-03, and finally to a supervisory system (Elipse SCADA™ v2.29).

The mapping of the pressure differential and PIG’s velocity was done through a neural network. The input variable is the pressure differential on the PIG and the output variable is the PIG’s speed. In this work, we used two different networks: a MLP and a NARX, both trained with the Levenberg-Marquardt algorithm [23].

The Levenberg-Marquardt algorithm uses the concept of backpropagation explained before, but its equation is an improvement of a basic backpropagation equation [21]. The Levenberg-Marquardt equation is presented below:

(7) $$\Delta w\left(t\right)={\left[J^{T}\left(t\right)\cdot J\left(t\right)+\lambda I\right]}^{-1}\cdot J^{T}\left(t\right)e\left(t\right),$$

where: *J* is the Jacobian matrix formed by the partial derivatives of the error e(t) in relation of the weights, *I* is identity matrix and λ is a learning rate regulation parameter. The improvement of this algorithm is due to the use of the Jacobian matrix and the regulation parameter λ, that leads to a reduced convergence time of the network in relation to others algorithms and its good training network results [21].

Due to the small size of data set, we used the crossover technique during training. In this technique, the data are randomly divided in r parts: r-1 parts are to the training step and one part is to validation step of the neural network. At each iteration, the parts of both groups are changed to ensure that each example is used in training and validation processes.

We used the Matlab™ Neural Network (r2015a) toolbox for training, validation and testing procedures. In addition, we used it to plot the data included in this paper. That software was run in a computer with Intel^®^ Core™ 2 Duo T6600 processor, 2.20 GHz, 4 GB RAM, Windows™ 7 32-bits operational system.

**Figure 8:**
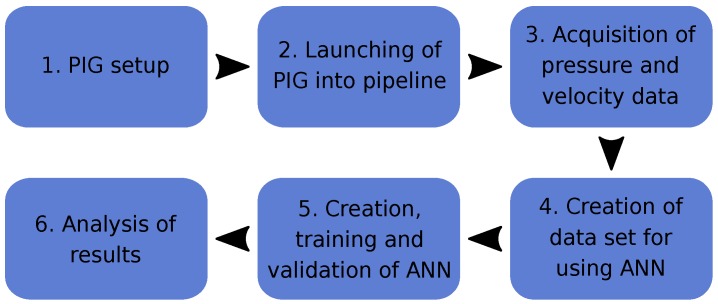
Flowchart of experimental procedure. Source: By authors.

In Figure 8, we present a flowchart that summarizes the experimental procedure for our research.

As indicated in Figure 8, the first step was the PIG setup (1), which consists of installing and turning on the electronic board in the PIG’s body. Then we inserted the PIG into the pipeline launcher and the pipeline is filled with compressed air (2). The embedded electronic board stored velocity data, based on the odometer, while the supervisory system stored pressure data from the transducers installed on the pipeline (3). When the PIG reached the end of the pipeline and it was depressurized, we recovered the PIG, opened it and collected its velocity data. We then treated the pressure data, stored by the supervisory system, and created the data set to be used in the artificial neural networks MLP and NARX (4). After the creation of the ANNs, they were trained and validated (5). Finally, we analyzed the results (6).

## 3. Results

**Figure 9:**
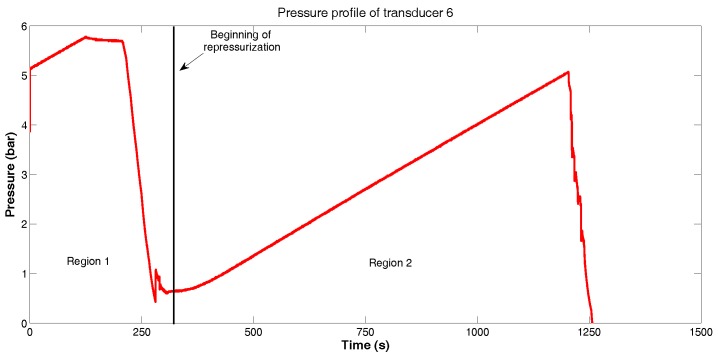
Pressure profile of transducer 6. Source: By authors.

During PIG’s test procedure, the pressure measurements of each sensor were taken at intervals of 50 ms. Pressure data obtained by the sensor 6 are shown in Figure 9. The same behavior was observed in other sensors.

As illustrated in Figure 9, the pressure data are divided in two regions: these regions indicate that the pipeline had to be repressurized because the pressure differential acting on PIG was not sufficient to promote its movement.

PIG’s velocity is directly proportional to the pressure differential on it. Table 1 presents the variables we have defined, which indicate the difference of pressure between two functional consecutive pressure sensors.

**Figure 10:**
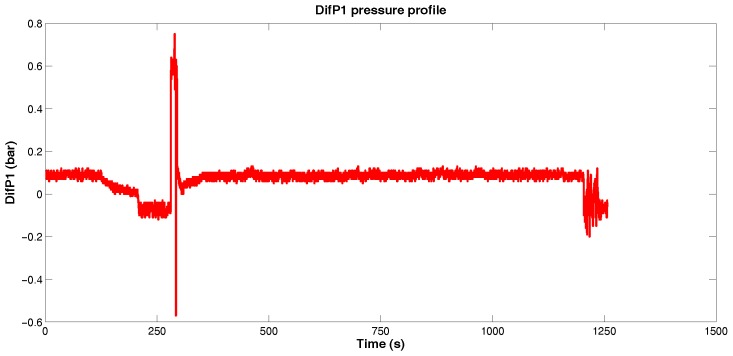
*DifP1* pressure profile. Source: By authors.

Figure 10 illustrates the variable *DifP1* along the PIG’s run in the pipeline.

**Figure 11:**
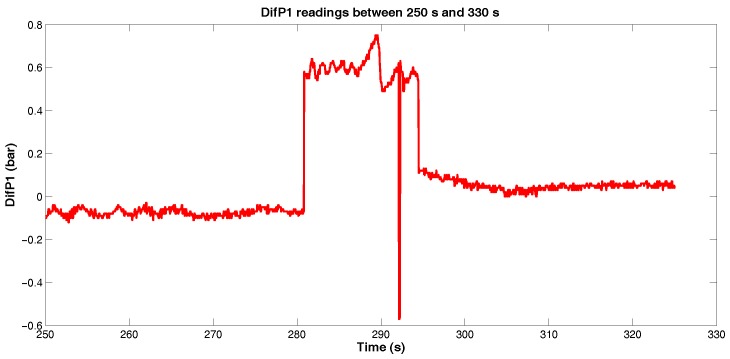
*DifP1* readings between 250 s and 330 s. Source: By authors.

**Figure 12:**
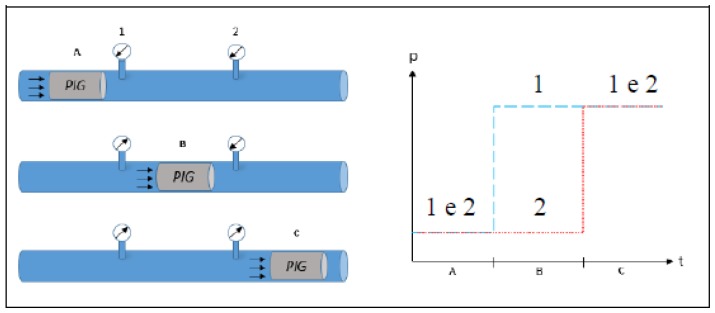
Effect of PIG on the readings of two consecutive pressure sensors. Source: From Freitas [15].

According to Figure 10, we can verify that the pressure differential between the sensors 6 and 5 remained between −0.2 bar and 0.2 bar, possibly associated with the fluid flow regime and the sensibility of sensors, but significant values were observed between 250 s and 325 s. Zooming this interval (Figure 11), we observe an increase of pressure differential between 280 s and 295 s, from approximately 0 bar to approximately 0.8 bar, which might indicate the passage of PIG along the sensors 6 and 5, as illustrated in Figure 12.

**Figure 13:**
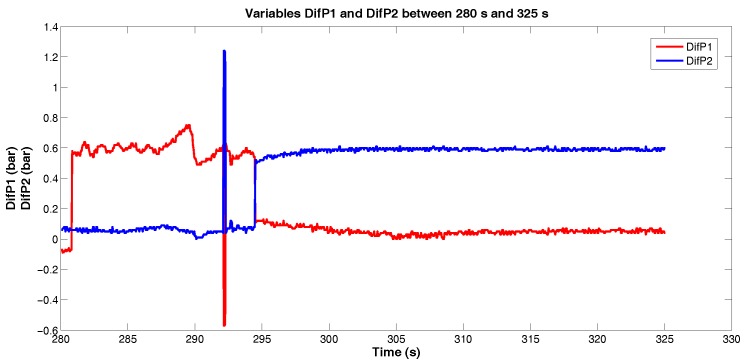
Variables *DifP1* and *DifP2* between 280 s and 325 s. Source: By Authors.

Doing this same treatment with the variable *DifP2* (Figure 13), a compliance with the variable *DifP1* results was observed, indicating the passage of the PIG between the sensors 5 and 4: the value of *DifP1* returned to zero and the value of *DifP2* increased.

From this point, right after the PIG comes in between sensors 5 and 4, the PIG stopped inside the pipeline due to an obstruction (defect on the pipeline or incrustation) or insufficient pressure differential to move the PIG.

**Figure 14:**
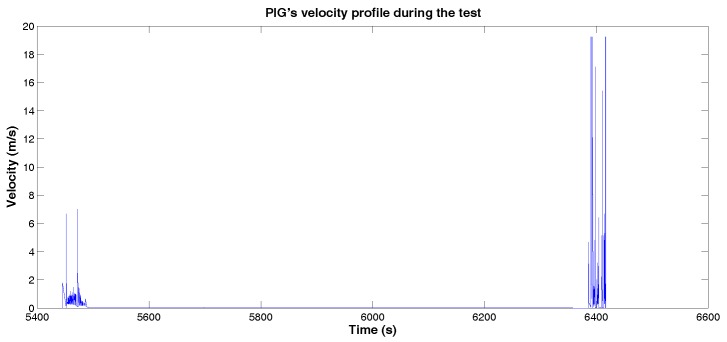
PIG’s velocity profile during the test. Source: By Authors.

Figure 14 shows the velocity profile of PIG’s test procedure. Like in Figure 9, it is possible to identify two moments during the test, divided in a moment to re-pressurize the pipeline and, consequently, without movement of the PIG.

**Figure 15:**
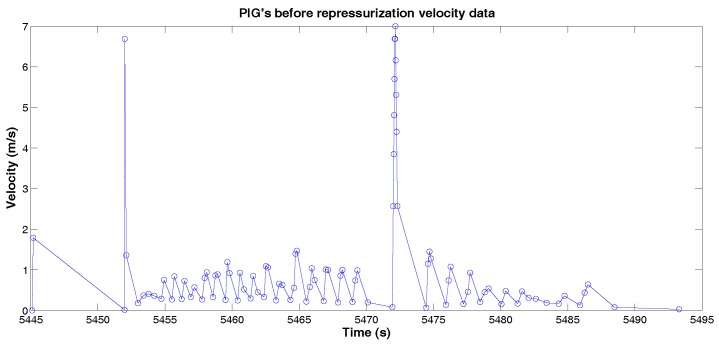
PIG’s before repressurization velocity data. Source: By Authors.

The PIG’s velocity before repressurization is presented in Figure 15. In this case, the average velocity was less than 2 m/s and the maximum velocity was 7 m/s.

**Figure 16:**
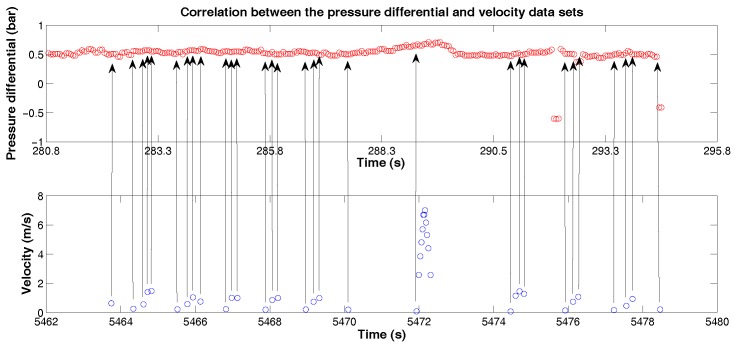
Correlation between the pressure differential and velocity data sets. Source: By Authors.

With the pressure differential and velocity profiles of the PIG, the next step was to correlate these two data sets (Figure 16).

In total, we obtained a new data set with 58 points correlated. From then on, we started the process of creation, training and validation of the MLP and NARX artificial neural networks, considering the pressure differential data as input and velocity data as output. Due to small size of dataset, we used the crossover technique, dividing the 58 points in 5 sets with 10 points each (for training) and one set with 8 points (for validation). One foreseen problem for the use of small data set at the training step is the poor generalization capability presented by the neural network when it has to predict new points. The crossover technique is able to provide a good response in training and validation for networks, since it divides the total data set in mini groups [21].

**Figure 17:**
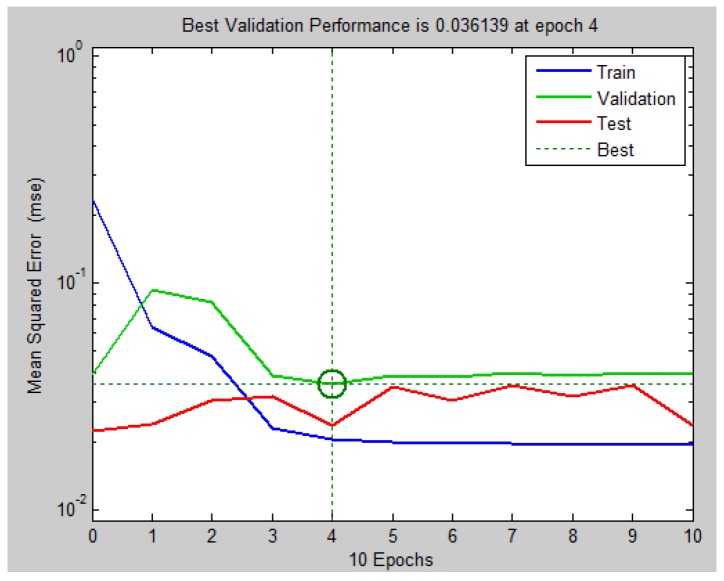
Result of validation for MLP neural networks created with structure 1:7:1. Source: By Authors.

Initially we created a MLP neural network with structure 1:5:1 (one input neuron, 5 neurons on the hidden layer and one output neuron), whose mean squared error obtained was 13.1% in validation step. Then, the number of neurons in the hidden layer was increased to six (structure 1:6:1), resulting in a mean squared error still above 5.0% in validation step, value targeted. When the number of neurons in the hidden layer was leveled up to 7 (Figure 17), the performance decreased to 3.6%, value of error acceptable. At this point, increasing the quantity of neurons did not result in significant increase of efficiency.

**Figure 18:**
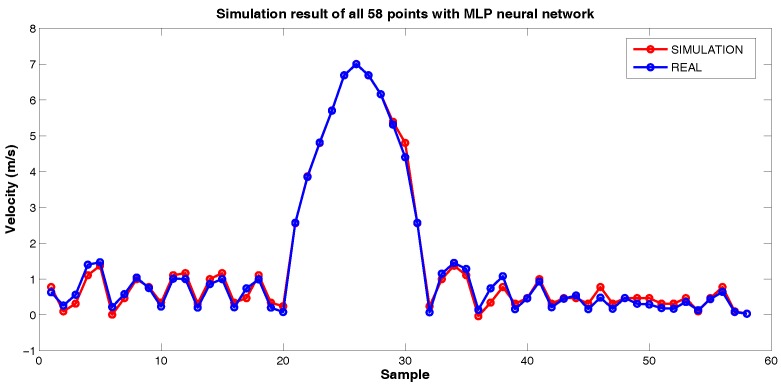
Simulation result of all 58 points with MLP neural network. Source: By Authors.

Then, it was used to simulate all 58 points (Figure 18), obtaining confirmation of the good result obtained in the validation and showing that it was possible to predict the velocity of the PIG using differential pressure data applied in a MLP neural network.

**Figure 19:**
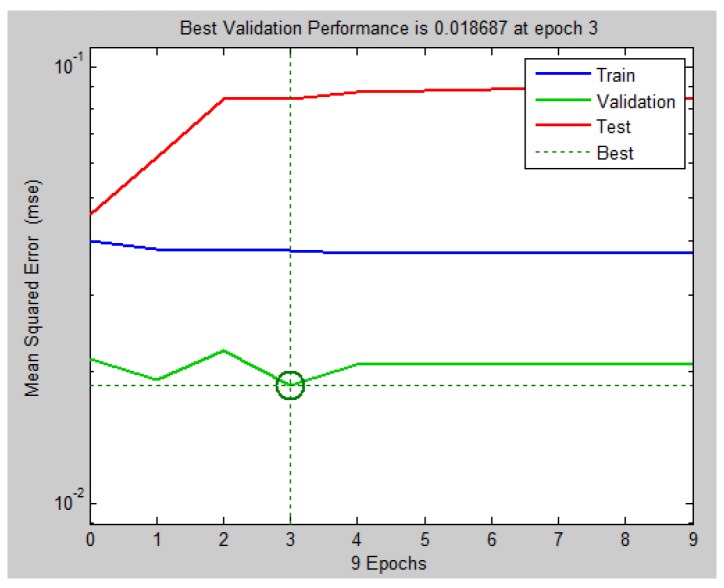
Result of validation for NARX neural networks created with 4 neurons in the only hidden layer. Source: By Authors.

The same procedure was applied for searching the best structure for the NARX model, but now the quantity of delay units in the input and output layers was also analyzed. Initially a structure with one delay unit in the input layer and two delay units in the output layers was investigated. Figure 19 illustrates the results achieved with a structure presenting four neurons in the sole hidden layer.

**Figure 20:**
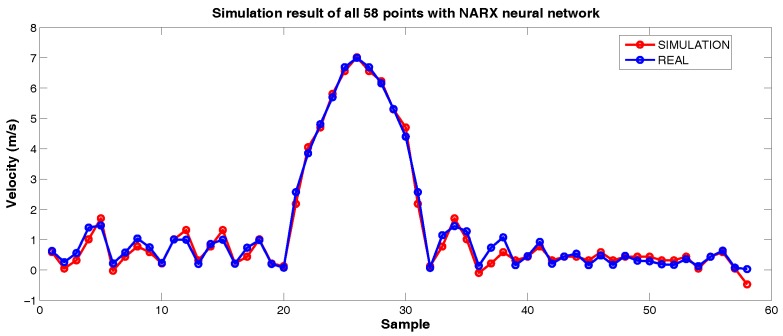
Simulation result of all 58 points with NARX neural network. Source: By Authors.

Figure 19 indicates that validation’s performance of NARX network was better than that obtained with MLP net (1.9% against 3.6%). It agrees with the theoretical result expected, that, in cases of time series modeling, a recurrent neural network is, in general, more appropriate [21]. Despite that, the MLP can achieve good results in some cases. Other structures were also tested, but as one of our further objectives is to implement the best neural network on the microcontroller (more complex structures require more computational cost) and the gains acquired were not significant, the selected structure was the one with 4 neurons in the hidden layer, one delay unit in input and two delay units in output. We present the simulation with all 58 points using NARX network in Figure 20, that indicates the net’s capability to predict the next velocity value from the pressure differential value.

## 4. Discussion

We found that it is possible to use neural networks to calculate the PIG’s velocity based on the pressure differential. We searched the best structure for each kind of artificial neural network based on simplicity of the structure and on its efficiency, so we used a low number of hidden layers and neurons for the MLP and NARX networks. For the NARX network, we used a low number of delay units in the input and output layers.

The most used method to obtain PIG’s speed is based on odometers. However, the odometer may present poor performance under several circumstances [13]. Therefore, we looked for an alternative way to obtain PIG’s velocity. According to Equation (5), the PIG’s velocity depends mainly on differential pressure, thus we could obtain a mathematical model that correlates these variables to predict velocity. Another approach, known as identification, is directly based on experimentation [24]. Among the several identication techniques, ANNs have been successfully employed in many applications, especially in non-linear systems [25,26,27]. However, the use of neural networks for calculating PIG’s velocity from pressure differential has not been previously addressed.

We think that the oil and gas industry, as well as other industries that use pigging, can benefit of our proposal to enhance the performance of the PIG’s velocity measurement. Operators (producers, transporters and other related functions) must do frequent inspections on its pipelines for identifying, through sensors, failures that diminish the flow of oil and gas products and sub-products inside. If the sensors do not operate between certain ranges of velocity, some parts of the pipe will not be inspected due to sensor failure. Furthermore, to compare the previous conditions to the current ones, operators keep data about the test of the pigging, such as pressures and velocity of the PIG along the pipeline during the entire test.

Since our current data set is small, we have applied the crossover technique for training the neural networks in order to minimize overfitting. We are presently working to collect more data, as further tests are required to verify the performance of our networks on new data.

The next step in this research is to embed pressure sensors on the PIG to investigate a way to obtain more accurate pressure values and, hence, more realistic velocity values. The embedded sensors will be used to provide us pressure data, which are currently provided by the supervisory system.

## 5. Conclusions

We have investigated the applicability of artificial neural networks, specifically MLP and NARX networks, to calculate the velocity of a smart PIG from differential pressure. The results indicate the possibility of acquisition of more reliable speed values, since the proposed system could be used as a redundant source of velocity data in cases of failures of primary speed sensors, like odometers. The reliability of the velocity values acquired with the ANN is potentially greater than using the odometer. We consider that the main contribution of this paper is to present the possibility of embedding an artificial neural network in a PIG in order to improve its velocity’s measurement system.

## Figures and Tables

**Table 1:** Description of pressure differential variables created.

Variable	Description
*DifP1*	Difference between transducers 6 and 5 readings
*DifP2*	Difference between transducers 5 and 4 readings
